# Fatal, Fulminant and Invasive Non-Typeable *Haemophilus influenzae* Infection in a Preterm Infant: A Re-Emerging Cause of Neonatal Sepsis

**DOI:** 10.3390/tropicalmed5010030

**Published:** 2020-02-20

**Authors:** Sudipta Roy Chowdhury, Srabani Bharadwaj, Suresh Chandran

**Affiliations:** 1 Department of Pediatric Medicine, KK Women’s and Children’s Hospital, Singapore 229899, Singapore; 2 Yong Loo Lin School of Medicine, National University of Singapore, Singapore 117597, Singapore; srabani.bharadwaj@singhealth.com.sg (S.B.); suresh.chandran@singhealth.com.sg (S.C.); 3 Lee Kong Chian School of Medicine, Nanyang Technological University, Singapore 639798, Singapore; 4 Duke-National University of Singapore Medical School, Singapore 169857, Singapore; 5 Department of Neonatal and Developmental Medicine, Singapore General Hospital, Singapore 169608, Singapore; 6 Department of Neonatology, KK Women’s and Children’s Hospital, Singapore 229899, Singapore

**Keywords:** non-typeable *Haemophilus influenza*, early-onset neonatal sepsis, preterm mortality

## Abstract

Early-onset neonatal sepsis (EOS) is a major cause of neonatal death and long-term neurodevelopmental disabilities among survivors. The common pathogens causing EOS are group B *streptococcus* (GBS) and *Escherichia coli*. *Haemophilus influenzae* (*H.* *influenzae*) is a Gram-negative coccobacillus that can cause severe invasive disease and can be divided into either typeable or non-typeable strains. *H.* *influenzae* serotype b (Hib) is the most virulent and the major cause of bacterial meningitis in young children prior to routine immunization against Hib. Hib infection rates have dramatically reduced since then. However, a number of studies have reported an increasing incidence of non-typeable *H. influenzae* (NTHi) sepsis in neonates worldwide and concluded that pregnant women may have an increased risk to invasive NTHi disease with poor pregnancy outcomes. We present a case of fulminant neonatal sepsis caused by NTHi in an extremely preterm infant and discuss potential preventative measures to reduce its re-emergence.

## 1. Introduction

*Haemophilus influenzae* usually colonizes the upper respiratory tract. Strains can be distinguished according to the polysaccharide capsule into six serotypes (a–f). Non-typeable *H*. *influenzae* (NTHi) do not have a polysaccharide capsule. Most *H*. *influenzae* strains are opportunistic pathogens and can potentially cause invasive infections in preterm infants [1].

Regular immunization against serotype b (Hib) has resulted in a sustained decrease in invasive Hib disease burden through direct and indirect protection [1,2].

As a result, in nations where there is mandatory or routine Hib immunization programs, NTHi has evolved as a more predominant type responsible for most invasive *H*. *influenzae* infections in newborn infants [1,3].

Furthermore, some studies have highlighted a significant burden of perinatal invasive NTHi disease, resulting in morbidity and mortality in pregnant women and newborn infants [1]. A Finnish study showed that neonates are at greater risk of invasive NTHi disease, suggesting a vertical transmission from their mother [4]. NTHi has been characterized as an invasive disease in preterm infants with significant rates of mortality and long-term morbidity. In preterm infants, mortality can be as high as 90%, especially amongst extremely preterm infants [1,4,5].

We report the emergence of fulminant NTHi sepsis in an extreme preterm infant in a tertiary neonatal unit of Singapore, our first report since the last two decades. This is the first reported fatal case of neonatal sepsis caused by NTHi in Singapore.

## 2. Case Presentation

The patient’s mother provided a written consent for the case report to be published. A 24-year-old healthcare staff (gravida 4, para 2) presented with preterm premature rupture of membranes (PPROM) at 27 weeks gestation. Her antenatal history was uneventful with unremarkable antenatal serology and fetal ultrasound scans. She was afebrile with no history of recent fever or upper respiratory tract infection symptoms. Fetal movements were reported to be normal. Examination revealed a rupture of the membrane but there were no evidence of vaginal bleeding, contractions or abdominal pain. She was not in threatened preterm labor.

She was admitted and prescribed oral erythromycin for antibiotic coverage for PPROM. Prior to this admission, she had a history of copious foul-smelling vaginal discharge for three weeks. Her hemogram on admission showed leukocytosis (16.99 × 10^9^/L) and raised C-reactive protein (max. 20.8 mg/L). Urinalysis was normal and urine bacteriological culture was sterile. Her high vaginal swab was positive for *Candida* species. Within four hours of admission, she developed fever (max. temperature 38.5 °C) and her cardiotocography (CTG) showed recurrent unprovoked decelerations with decreased fetal movements. She went in for an urgent lower segment caesarean section, indications being non-reassuring fetal status and meconium stained liquor.

A female infant weighing 1000 g was delivered with poor respiratory effort and Apgar scores of 3 and 5 at one and five minutes, respectively. She received full neonatal resuscitation and was intubated and transferred to the neonatal intensive care unit (NICU). Endotracheal surfactant was administered for respiratory distress syndrome of prematurity. There was very minimal response to surfactant administration and her respiratory status continued to deteriorate. Her first arterial blood gas at one hour of age showed marked metabolic acidosis with severe hypoxia. Her ventilation was escalated to high frequency oscillatory ventilation (HFOV) with inhaled nitric oxide at 20 ppm (parts per million) in view of persistent hypoxemia. She was hypotensive and received fluid boluses and multiple inotropic supports. Intravenous antibiotics, ampicillin at 100 mg/kg and gentamicin at 2.5 mg/kg, were administered for presumed sepsis after blood was sent for full blood count and blood cultures. She also received intravenous sodium bicarbonate for correction of severe metabolic acidosis. A chest X-ray was performed and surface swabs and tracheal aspirates were tested for possible aerobic cultures. A cranial ultrasound scan was also performed to rule out any intracranial bleeds.

The full blood count revealed bicytopenia (hemoglobin 8.2 g/dL, total white cell counts 6 × 10 ^9^/L and platelets 89 × 10^9^/L). Her cranial ultrasound scan was normal with no intraventricular or intracerebral haemorrhage. The chest radiograph showed bilateral coarse reticulo-granular markings and an air bronchogram with no signs of air leak or hyperinflation. After 24 h, the infant’s blood and tracheal aspirate cultures were tested positive for NTHi that was sensitive to ampicillin, augmentin and ceftriaxone. Maternal placental swab cultures also revealed NTHi with the antibiotic susceptibility that is likely to be the same strain with histopathological evidence of chorioamnionitis. Thus, the transmission of NTHi was deemed to be vertically transmitted and could have originated from the mother’s vaginal discharge.

Despite intensive-care support and management, the infant had further clinical deterioration with profound hypotension and eventually became asystolic. There was no return of spontaneous circulation even after 20 min of effective resuscitation. The infant died at approximately four hours of life.

## 3. Conclusions

For the first time in two decades, we report a fatal cause of neonatal sepsis attributed to NTHi in an extremely preterm infant in Singapore. With reduction of incidence in Hib infection worldwide, especially in countries with mandatory Hib immunization, there has been a reported rise in the incidence of the other strains of *H*. *influenzae.* Routine vaccination for Hib was introduced in Singapore in 2013 though it was being administered privately. A previous study done reported an Hib incidence rate of 0.57 per 100,000 amongst <5-year-old children (January 2004–December 2012), which showed an 87% reduction when compared to a reported incidence of 4.4/100,000 during the period of 1994–2003 [2].

Invasive NTHi infection in neonates has been reported in the last few years in the literature worldwide. The mortality rate appears to be as high as 90%, especially in extreme premature infants who are less than 30 weeks gestational age [6,7]. Both NTHi- and GBS-associated sepsis have a similar risk profile in many ways, with the infected infants more likely to be preterm with maternal risk factors of maternal fever, prolonged rupture of membranes, and evidence of clinical and biochemical chorioamnionitis [8]. Collins et al. reported that though only around 1.8/1000 pregnant women carry NTHi in their genital tract; the prevalence was higher (7.3%) in women with PPROM. This is in contrast to GBS, which commonly colonizes the reproductive tract during pregnancy but only occasionally cause invasive disease in the newborn. Despite this, it is still important to highlight that NTHi might be emerging, but GBS is still more common as a cause of neonatal sepsis.

The mother of our index case was a healthcare worker. She was more vulnerable to exposure to upper respiratory *H*. *influenzae* strains though there is speculation that genital tract NTHi strains are different from those colonizing the nasopharynx and this warrants further research [9].

The mother of our infant gave a history of prolonged vaginal discharge, which could be an indicator of chorioamnionitis, it may be advisable to empirically start prophylactic broad-spectrum intravenous antibiotics.

This is the first report of a fatal case of neonatal sepsis caused by NTHi in Singapore. Over the past 20 years, there has been no reported case of NTHi-related neonatal infection in all three tertiary neonatal centers in Singapore. Since NTHi has not been historically associated with neonatal sepsis, none of our tertiary centers has specific antimicrobial guidelines in managing this infection. The prevalence/impact of NTHi may have been under-reported. Both the fastidious nature of the *Haemophilus* genus and the requirement of special blood agar medium for its growth could have contributed to this occurrence. Thus, it may not have been isolated in many cases and were under-reported in culture-negative EOS.

NTHi is often identified as a normal commensal in the upper respiratory tract of children and commonly only causes respiratory tract diseases. However, neonatal infections have been linked to colonization of the maternal genital tract [9]. Although there was histopathological definite evidence of chorioamnionitis, we did not detect NTHi from maternal vaginal culture in our index case. NTHi was only identified when it was present in moderate to large quantities [9]. Thus, infections with low colony counts may remain undetected as assumed in our case.

Because of the high mortality risks associated with NTHi, this emerging pathogen that causes neonatal sepsis, especially in preterm neonates, should be re-evaluated and taken seriously. Studies show evidence of a 10-times increased risk of invasive NTHi disease compared with Hib during the first week of life, thus our focus should be now shifted to this type [10]. It is therefore important for both obstetricians and neonatologists to be aware of its increasing potential risk. NTHi has been an under-recognized pathogen and, therefore, we hope to generate more awareness on its disease burden in the perinatal fraternity. NTHi was previously thought to be a commensal. Nonetheless, recent evidence is pointing towards increased neonatal morbidity and mortality. Thus, as physicians, we need to pay closer attention.

In conclusion, our case report shows that NTHi is a potential maternal, fetal and neonatal pathogen. We recommend that in suspected cases, the culture medium should be specific to ensure accurate detection of any *H*. *influenzae* colonies. Any growth of *H*. *influenzae* in maternal vaginal swabs or placental swabs must be promptly highlighted to the requesting clinician and attending neonatologist. It will be of utmost importance to initiate appropriate antibiotics even to an asymptomatic neonate to prevent such devastating mortality or morbidity.
